# Perceptions on barriers, facilitators, and recommendations related to mental health service delivery during the COVID-19 pandemic in Quebec, Canada: a qualitative descriptive study

**DOI:** 10.1186/s12875-022-01634-w

**Published:** 2022-02-21

**Authors:** Jessica Spagnolo, Marie Beauséjour, Marie-Josée Fleury, Jean-François Clément, Claire Gamache, Carine Sauvé, Lyne Couture, Richard Fleet, Shane Knight, Christine Gilbert, Helen-Maria Vasiliadis

**Affiliations:** 1 Université de Sherbrooke, Sherbrooke, Canada; 2 Centre de recherche Charles-LeMoyne, Université de Sherbrooke, Campus Longueuil, Longueuil, Canada; 3 Douglas Mental Health University Institute, Montreal, Canada; 4 McGill University, Montreal, Canada; 5 CISSS de la Montérégie-Est, Saint-Hyacinthe, Canada; 6 CISSS de Laval, Laval, Canada; 7 CISSS de la Montérégie-Centre, Taschereau, Canada; 8 CIUSSS du Centre-Sud-de-l’Ile-de-Montréal, Montreal, Canada; 9 Université Laval, Quebec City, Canada; 10 Initiative Patients-Partenaires, Université de Sherbrooke, Sherbrooke, Canada

**Keywords:** COVID-19, Mental health, Service delivery, Primary care, Quebec, Canada

## Abstract

**Background:**

There was an increase in self-reported mental health needs during the COVID-19 pandemic in Canada, with research showing reduced access to mental health services in comparison to pre-pandemic levels. This paper explores 1) barriers and facilitating factors associated with mental health service delivery via primary care settings during the first two pandemic waves in Quebec, Canada, and 2) recommendations to addressing these barriers.

**Methods:**

A qualitative descriptive study design was used. Semi-structured interviews with 20 participants (health managers, family physicians, mental health clinicians) were conducted and coded using a thematic analysis approach.

**Results:**

Barriers and facilitating factors were organized according to Chaudoir et al. (2013)‘s framework of structural, organizational, provider- and patient-related, as well as innovation (technological modalities for service delivery) categories. Barriers included relocation of mental health staff to non-mental health related COVID-19 tasks (structural); mental health service interruption (organizational); mental health staff on preventive/medical leave (provider); the pandemic’s effect on consultations (i.e., perceptions of increased demand) (patients); and challenges with the use of technological modalities (innovation). Facilitating factors included reinforcements to mental health care teams (structural); perceptions of reductions in wait times for mental health evaluations during the second wave due to diminished FP referrals in the first wave, as well as supports (i.e., management, private sector, mental health trained staff) for mental health service delivery (organizational); staff’s mental health consultation practices (provider); and advantages in increasing the use of technological modalities in practice (innovation).

**Conclusions:**

To our knowledge, this is the first study to explore barriers and facilitating factors to mental health service delivery during the pandemic in Quebec, Canada. Some barriers identified were caused by the pandemic, such as the relocation of staff to non-mental health services and mental health service interruption. Offering services virtually seemed to facilitate mental health service delivery only for certain population groups. Recommendations related to building and strengthening human and technological capacity during the pandemic can inform mental health practices and policies to improve mental health service delivery in primary care settings and access to mental health services via access points.

**Supplementary Information:**

The online version contains supplementary material available at 10.1186/s12875-022-01634-w.

## Background

Studies have highlighted the negative consequences of the COVID-19 pandemic on population mental health, showing increasing trends of self-reported anxiety and depression symptoms in Canadian adults [1, 2], and this across provinces [2, 3]. Despite this evidence, 20% of Canadians reported consulting healthcare professionals for mental health reasons as opposed to one third, prior to the pandemic [2]. Most significant decreases were for in-person consultations with a mental health professional (from 23 to 6%) and mental health consultations with a family physician (FP) (from 12 to 6%) [2].

Research conducted during the pandemic shows barriers to mental health care in Canada, and this in the context of already existing challenging access to mental health care [4]. In Ontario, Canada, such barriers during the pandemic included increased wait time for services, challenges with virtual care (e.g., difficulties in establishing therapeutic rapport, limited training offered to providers, patients without access to technology, obstacles in transitioning groups online), and increased workload for mental health providers [5, 6]. Facilitators to mental health service delivery were also noted and included increased accessibility to services for certain patients via virtual care [5], stepped care approaches to mental health care delivery, and the involvement of primary care practitioners/teams in the provision of mental health services [5–7]. Pandemic barriers and facilitators to mental health service delivery are to our knowledge currently unknown in Quebec, the Canadian province with the highest rate of COVID-19 mortality (September 21st, 2021) [8] and the only province with an implemented nightly curfew [9], challenges that may have impacted population mental health.

In Quebec, like in the rest of Canada and many countries worldwide, primary care settings are important access points for mental health service delivery [10–13]. Family physicians (FPs) working in primary care are often the first access point for mental health care [11, 14] and are therefore important to include in discussions around mental health service delivery in the context of the pandemic [5, 15]. Non-physician perspectives like those of staff working at mental health referral mechanisms in primary care settings are important to better understand mental health service delivery in the pandemic, as they have also been less studied in this context [5, 6]. In Quebec, two mental health referrals mechanisms are available and used by FPs: i) single regional access points (*Centres de répartition des demandes de services* (CRDS)) for processing requests to specialized health services for a first time consultation with specialists, including specialized psychiatry since 2019 [16–18]; ii) *Guichets d’accès en santé mentale adulte* (GASMA), implemented since 2008 and widely used local mental health service access points for patient mental health evaluation and referral to appropriate local mental health services (psychosocial and/or psychiatric) according to evaluated needs [10, 19–21]. The CRDS was implemented as part of a program to improve access to specialized health services, including access to psychiatrists. Specifically, FPs could fill out and submit requests for patient referrals to psychiatrists using a standardized form with predefined levels of prioritization according to the listed reasons for consultation. This form would then be submitted to the centralized referral system, where specialists including psychiatrists were assigned [18]. The GASMA is a referral mechanism used by FPs and other healthcare professionals, who judge that patients might require community or primary mental health care, or psychiatric services (offered by psychiatrists). Level of care and healthcare professional for referral is determined after patients have been evaluated by GASMA health professionals [10, 19–21].

Recommendations were produced to help health establishments, including these referral mechanisms, with health care organization and mental health service delivery during the first and the second pandemic waves [22–24]. They were produced by the Quebec Ministry of Health and Social Services and published for public access in the form of an accompanying information and staff relocation guide [22–24]. For example, some ministerial recommendations specifically addressed anticipated increases in demands for mental health care, and included 1) the use of stepped care (e.g. directed and self-directed self-care), increased collaboration between community-based, primary, and specialized care, and increased involvement of community-based settings in patient care when appropriate; 2) a follow-up from the GASMA teams with people whose mental health consultations may have been postponed/or who are waiting for services; and 3) the prioritization of virtual group and individual consultations, and if group content could not be adapted virtually, alternatives were to be discussed (e.g., individual meetings or implementing self-care strategies) [22–24]. Mental health interventions were allowed in-person should providers judge it necessary to identify symptoms related to mental health conditions that may otherwise be missed by teleconsultations [24]. The Ministry recommended that GASMA mental health evaluation teams would receive support from other teams if staffing became an issue, and this to ensure that new requests for mental health services were processed in a timely manner [22, 23]. Specifically, in November 2020 (during Quebec’s second wave), the Ministry mentioned the essentiality of mental health services and recommended against the relocation of mental healthcare staff [24]. In addition, specialized services would prioritize people at higher risk (e.g., with depression, anxiety disorders, and psychosis, and/or a danger to themselves/others), as well as with co-morbidities, cognitive disorders, and behavioural issues [22].

The study objectives were to explore from the perspective of stakeholders (healthcare professionals working in mental health including nurse practitioners, nurse liaisons, social workers, family physicians, psychiatrists, as well as health managers): 1) the barriers and facilitating factors related to mental health service delivery via primary care during the first two waves of the pandemic in Quebec, and 2) the recommendations to address these barriers. Findings can inform on solutions that may be useful for mental health practice and policy during and post-pandemic in Quebec, Canada, and other countries where primary care is an important first point of access for mental health care.

## Methods

### Study design and setting

To meet the research objectives of this paper, we relied on a qualitative research design [25, 26], which “provides a vehicle for the voices of those experiencing the phenomena of interest” [25] to explore barriers and facilitating factors to mental health care delivery during the first two pandemic waves in Quebec, and this from the perspectives of key actors like healthcare professionals working in mental health (nurse practitioners, nurse liaisons, social workers, family physicians, and psychiatrists), as well as health managers. Wave 1 occurred between March and May 2020, and wave 2, between September 2020 and March 2021 [27].

This paper includes findings from three Quebec healthcare university and peripheral regions [28–30]. The peripheral regions of the study include *Centres intégrés de santé et de services sociaux* (CISSS) (English: Integrated Health and Social Services Centres): hospital centres, clinics, group homes, child protection centres, and rehabilitation centres. The healthcare university regions include all services of the CISSS that are located in a region that has a university which offers a full undergraduate medical program and/or operates a centre designated as a university affiliated institute in the health and/or social fields. The healthcare university study region is therefore classified as *Centres intégrés universitaires de santé et de services sociaux* (CIUSSS) (English: Integrated Health and Social Services University Centres). These three regions were selected for a larger research project based on diversity of service supply and health care system organization characteristics to better understand the implementation, functioning, and use of the CRDS and its complementarity with the GASMA. These three regions group 4,073,950 habitants out of Quebec’s total population of 8,447,000 habitants (2019). Population density in the three regions ranged from 1710.9 to 4121.1 habitants/square kilometre. Each region has one operating CRDS. The healthcare university region and one of the peripheral regions have multiple GASMA, whereas the other peripheral region has one GASMA. Of note, the study regions had one of the highest COVID-19 pandemic related outcomes. As of 21 September 2021, the three regions had some of the highest numbers of COVID-19 deaths in Quebec, and the highest rates of confirmed cumulative COVID-19 cases since the start of the pandemic [31].

### Study participants

Participants were recruited in two ways: 1) purposeful sampling, where potential participants were identified for their knowledge about mental health service delivery via the CRDS and/or the GASMA [32]; and 2) snowball sampling, useful “for locating information-rich key informants” [32]. For purposeful sampling, we recruited participants through internet searches and through our networks in the study regions. These networks stem from our research team, as well as from a previous project on the functioning and use of the CRDS for all 26 specialties [18]. For snowball sampling, JS asked participants identified through purposeful sampling if they knew of any key informants potentially interested in the research. Participants were contacted by JS using a personalized email which explained the study and participant role. If the individual confirmed interest, JS sent by email the consent form which included additional information about the study and scheduled a telephone interview. Twenty individuals participated in the research. This study received ethics approval from the Institutional Review Board of the *CIUSSS de l’Estrie – Centre hospitalier universitaire de Sherbrooke* (CHUS) (English: Sherbrooke University Hospital Center) (MP-31-2020-3662), and from the Institutional Review Boards of the three study regions. All study subjects were presented the study objectives and what their participation involved. Informed verbal and written consent were obtained. No honorarium was provided for participation in the interview.

### Data collection

Data was collected through semi-structured individual interviews between November 2020 and June 2021. An interview guide with open-ended questions was developed for the purposes of a larger research project, and it included questions on barriers and facilitators to mental health service delivery during the pandemic. The guide was reviewed by the research team, which includes researchers, clinicians (FP and a psychiatrist), health managers, and patient partners. Interviews were conducted in French by JS and transcribed verbatim.

### Data analysis and scientific rigor

Interview transcriptions were coded using a thematic analysis approach [33], consistent with data analyses for the descriptive qualitative design [25, 26]. For this paper, solely verbatim that centred around mental health service delivery during the pandemic context was coded. Specifically, this paper is part of a larger research project aiming to understand the functioning and use of the CRDS specifically for psychiatry, and its complementarity with other referral mechanisms like the GASMA. Given that this project was conducted during the COVID-19 pandemic, we were able to include a section on mental health service delivery including via these referral mechanisms during the COVID-19 pandemic. In a first step, JS read all the transcriptions prior to coding as a refamiliarization process and to highlight the COVID-19 related passages in NVivo 12 Pro software. In a second step, JS coded using NVivo 12 Pro software broad passages into overarching themes that she developed to reflect them, following an inductive approach [33]. In a third step, JS reviewed these broad passages assigned to the themes to further develop smaller units of analysis like categories and codes [33]. In a fourth step, this process was discussed with authors MB and HMV. Codes were verified to ensure validity (multiple examiners [34]) and questions to clarify certain codes and examples were noted to inform a knowledge sharing meeting with the research team. Also during this fourth step, JS organized findings on barriers and facilitating factors to mental health service delivery, as well as recommendations according to the following factors (where applicable): structural (ex.: socio-political context in Quebec); organizational (ex.: healthcare organization characteristics); provider (ex.: healthcare provider characteristics); patient (ex.: patient profiles, as well as health beliefs); and innovation (ex.: characteristics of newly implemented programs/interventions – in this case technological modalities for service delivery) [35]. These categories have been hypothesized to influence healthcare delivery and are part of a multi-level framework [35]. Participant quotes in the results section were translated from French into English by JS.

## Findings

Participants (*n* = 20) included: health managers working at the CRDS (*n* = 3) and chiefs of service in mental health (i.e., managers who oversee mental health initiatives and services) (n = 2); 8 mental health clinicians, including 4 healthcare professionals working at the GASMA and 4 psychiatrists; and 7 FPs working in university family medicine groups (a group of doctors working in an establishment, also with nurses) or clinics. Four participants were recruited from Region A, 11 from Region B, and 5 from Region C.

### Barriers and facilitating factors to mental health service delivery

Participants shared barriers and facilitating factors to mental health service delivery during the pandemic’s first two waves, and these are summarized in Table 1. These barriers and facilitating factors including illustrative examples are elaborated below.

**Table 1:** Summary of barriers and facilitating factors to mental health service delivery during the pandemic

Factors	Barriers	Facilitating factors
**Structural**	**1. Ministerial directives during the pandemic** • mental health staff relocation to *“COVID red zones”* • treating emergency cases only **2. Mental health staff shortage** • difficulties in recruitment for mental health care/limited number of mental health staff during the pandemic • limited availability of psychiatrists and psychologists during the pandemic • reduced staff at mental health access points	**1. Ministerial directives during the pandemic** • reinforcements to mental health care teams by relocating mental health staff to offer/reinforce mental health care **2. Learning from the pandemic’s first wave** • new funding for mental health teams • implementing and mobilizing technological modalities for mental health service delivery • new knowledge about the virus informing hygiene practices to facilitate re-opening clinics
**Organizational**	**1. Mental health service interruption** • community-based care (groups in community settings used by psychiatrists to address patient isolation) • primary care settings (family physician (FP) clinic closures, group interruption) • hospital outpatient clinics (closures, group therapy interruption)	**1. Reduced delays** • *Guichets d’accès en santé mentale adulte* (GASMA) (local mental health service access points) wait time to evaluate patient requests for services decreased **2. Support in the provision of mental health care, including through collaborations** • managerial support (e.g., facilitating the transition to technological modalities; allowing for staff to see certain patients in-person when there were limitations to technological modalities; offering lunch-time webinars and discussions for physicians on how to help with limited resources in a crisis context; *“officializing”* the use of mental health services in the private sector for *“more fortunate”* patients) • inter-organizational collaborations through availability of a social worker, psychologist, and nurse practitioner at the FPs’ health establishment • inter-organizational collaborations through meetings between all chiefs of services from the medical sector including mental health to coordinate health service delivery during the pandemic, as well as a community of practice for FPs working in substance use
**Provider**	**1. Mental health staff on leave** • COVID-19 preventive measures • medical leave (mental health related) **2. Physician availability and provider mental health capacity** • less in-person FP clinical activities • FP feeling *“alone”* for mental health care • psychiatrists with dual role of clinician and mental health manager during the COVID-19 context	**1. Practice characteristics adopted by healthcare professionals** • care and follow-up for “unattached patients” (patients without an FP) • GASMA workers contacting patients on wait lists to provide support and/or referrals to community organizations • *“reaching out”* to more vulnerable patients by developing/mobilizing community resources
**Patient**	**1. Pandemic’s effect on consultations** • patients not consulting during the first wave given the fear of the virus and because they thought the pandemic would be short-lived • *“people who have always worked well, who have always adapted well”* consulting, as well as people with certain vulnerabilities • the pandemic’s impact on everyone, but additional impact on people with certain vulnerabilities • the pandemic’s impact on people’s mental health, a new subject in FP assessment and discussions for the management of clinic waitlists	–
**Innovation (technological modalities for mental health service delivery)**	**1. Technological modalities for mental health service delivery** • inability to capture certain information necessary to evaluate patients and/or provide care (for staff) • shift to teleconsultations for mental health service delivery and its impact on certain patients’ access to mental health care (no email address, no technology access) (for patients)	**1. Technological modalities for mental health service delivery** • the pandemic *“propelling”* Quebec into *“computerization”* • *“catching up”* on consultations • satisfaction with and utility of technological consultations (e.g.: efficiency (seeing patients quicker, patients not being late), useful for patients with certain socio-demographic and clinical characteristics)

#### Structural barriers

##### Ministerial directives during the pandemic

CRDS staff were relocated during the first pandemic wave to Quebec *“COVID red zones”* so as to promote *“a shedding of activities [ …*] *to refocus on hospital activities and basic necessities”* (health manager 3). GASMA workers were reassigned to other clinical tasks such as work in residential and long-term care facilities for the elderly (outside of the mental health field), or hospital outpatient clinics to replace colleagues who were transferred to a Quebec *“COVID red zone.”* In the context of relocating staff, another pan-Quebec ministerial directive put forth related to treating urgent cases only. The reorganization of services contributed to making mental health resources even scarcer, including in healthcare organizations that were considered *“well staffed”* during the pandemic (psychiatrist 1).

##### Mental health staff shortage

Challenges related to the number of available mental health care staff were highlighted as a barrier to mental health service delivery. Participants shared that difficulties in recruiting social workers and nurses in the mental health care sector related to the unattractiveness of the field and the difficult issues surrounding the clientele, a barrier aggravated by the pandemic: *“basically, [even] without COVID, it’s not an attractive field for people, [they] are very scared of mental health, it is not an easy clientele and with COVID, it is even worse”* (GASMA 2). Participants also mentioned the limited number and availability of psychiatrists, including to conduct GASMA patient evaluations during the pandemic and at hospital outpatient clinics. In addition, participants highlighted the limited number of psychologists. At some GASMA points of access, reduced staff and/or their availability brought on challenges to its operation, leading to increased delays in patient evaluations and referrals to psychosocial and/or psychiatric services.

#### Organizational barriers

##### Mental health service interruption

Service interruptions were seen in community-based and primary care settings, as well as in psychiatric care. The pandemic halted groups in community settings, used by psychiatrists to address patient isolation, as well as groups that the GASMA referred to in primary care settings such as a newly implemented stepped care program to encourage self-care first through self-management (*Programme québécois pour les troubles mentaux, PQPTM*). Service interruptions were also seen in primary care clinics: *“some medical clinics were closed”* and *“there were health institutions [university family medicine groups] that were transformed into COVID clinics”* (GASMA 3). These closures, according to participants, reduced accessibility to FPs. Hospital outpatient clinics were closed during the first wave, a service interruption that prevented psychiatrists from seeing in consultations new patient requests from the GASMA, and this for several weeks, contributing to increased wait times. Group therapy for anxiety, personality, and bipolar disorders was also suspended at these clinics, including during the second wave. Given the limited number of mental health resources, as participants reported, replacing cancelled group therapy with individual sessions was not feasible.

#### Provider barriers

##### Mental health staff on leave

Participants shared that it was not uncommon for mental health staff to be on preventive (confinement related to the SARS-CoV2 infection) and medical leave. Preventive leave lasted for 2 weeks and increased workload burden on remaining staff. Participants also shared that it was more common during the pandemic for mental health staff including at the GASMA to be on medical leave for mental health issues as opposed to physical issues prior to the pandemic: *“before [the pandemic], the cause of leave for first-line workers was more for physical problems [ …] [but] since COVID, there is a lot more burnout, exhaustion at work than before”* (GASMA 2).

##### Physician availability and provider mental health capacity

GASMA staff highlighted that FPs were less available for in-person clinical activities due to clinic closures or their transformation to COVID-19 clinics during the first pandemic wave. There was also a need for increased FP involvement in mental health care delivery during the pandemic context, but *“some [were] not necessarily comfortable doing so”* (FP 6). Limitations to mental health capacity were seen among other healthcare providers. An FP shared that the clinic in which she works has access to a nurse practitioner, who works with physicians to treat most health conditions. However, collaborations are challenged for mental health care given that this nurse practitioner does not have expertise in mental health, leaving the FP to feel alone for the treatment of patients needing mental health care.

There were also challenges for health managers wearing both a COVID-19 planner and clinician hat. During the first pandemic wave, there were regular meetings with Ministry representatives and different regional sector representatives, to develop COVID-19 plans and to standardize processes at the regional level. These administrative tasks added to medical practice responsibilities and significantly increased their workload.

#### Patient barriers

##### Pandemic’s effect on consultations

According to participants, people consulted FPs less during the first wave, including out of fear of the virus and because *“people thought [the pandemic] would be short, they waited for it to pass”* (GASMA 2). However, perceptions were that there was more demand for mental health services via the CRDS and the GASMA during the second wave. In this context, participants reported a wide spectrum of people consulting for mental health services, from *“people who have always worked well, who have always adapted well”* (health manager 5) to people who already had a certain level of vulnerability prior to the pandemic. This vulnerability was described by participants as being related to certain socio-demographic and/or clinical characteristics: patients who were isolated/lived alone; people living with mental health disorders, adjustment disorders, and cognitive disorders; people experiencing homelessness; and people experiencing domestic violence. According to participants, mental health service delivery for certain people already experiencing some level of vulnerability was challenging. A health manager shared that *“in the first wave [ …] people with mental health issues didn’t dare step outside, they were very fearful [and] this clientele remains very fearful of what is going on”* (health manager 4). In addition, a psychiatrist from Region A shared that he noticed patients with psychosis missing their appointments for prescription re-fills. Other types of ‘new’ patient profiles were mentioned, including working parents with children, as well as primary and secondary school students, those enrolled in university and young professionals. According to participants, job loss, school closures, limited socialization/activities, and/or confinement interfered with healthy habits and defence mechanisms, prompting the need to consult for psychosocial support. With the pandemic’s effect, there is now *“one more thing to evaluate at every consultation [ …] so that adds to the assessment, [ …] to make sure that nothing is overlooked”* (FP 2). These cases were also discussed in meetings when managing clinic waitlists for access to psychosocial services: *“[ …] administratively, we talk a lot about targets, but I always want us to keep in mind that there is a user at the end of that as well [and the] faster we meet their needs, well the better it is, the less the situation is likely to deteriorate and get worse”* (health manager 5).

#### Innovation barriers (technological modalities for service delivery)

##### Technological modalities for mental health service delivery

Technological modalities for care (i.e., phone/video sessions with providers instead of face-to-face consultations) may miss important patient information, noticeable mainly through non-verbal cues during face-to-face consultations: *“we don’t have the non-verbal, how is the patient, is he neglected, not washed [ …] I think we have a better idea when we see the patient in person, for cases that are going badly and for those that are new”* (FP 5). Patients of a certain age or patients who did not have an email address and/or material resources to access web platforms were at a disadvantage with this type of care modality.

#### Structural facilitating factors

##### Ministerial directives during the pandemic

Health managers were invited to prioritize mental health service access points during the pandemic context given the growing demand for services and the need to maintain timely access to appropriate care. One example of this includes asking mental health workers to relocate to mental health access points: “*I had a position in psychiatry [ …] the fact that I am not currently in psychiatry but at the GASMA is a way that our program manager decided to support the GASMA, because [ …] we want to maintain the response times”* (GASMA 2). In addition, less solicited staff (ex.: social workers) at hospital outpatient clinics in Region A were made available for occupational or support therapy 2 hours a day, and this to hospitalized patients for mental health conditions in isolation for up to 14 days.

##### Learning from the pandemic’s first wave

Other successes at the structural level included increased funding to support mental health teams and learned lessons from the pandemic’s first wave, like the importance of implementing virtual care to continue seeing patients in consultations: *“In wave 1, [ …] we didn’t have the technology, it wasn’t deployed, and we had a big drop in activities, [ …] and now in phase 2, we are ready because we have deployed this tool and we are able to run at 100% of our capacity and even a little more in times of pandemic [ …] the only difference we have is, well apart from the preparation, is that we have the knowledge, the telemedicine, and that we are able to use it”* (health manager 3). Participants also shared the importance of learning more about the virus and public health measures (e.g., disinfecting chairs, wearing masks, making sure patients do not arrive more than a certain amount of time before their consultation, etc.) to conduct in-person consultations, specifically with new patients. This knowledge in wave 2 ensured that *“the outpatient clinic was not closed at all”* (psychiatrist 3). Given lessons learned from the first wave, participants shared that mental health services were better utilized, and staff were more organized during the second wave. Health managers with clinical responsibilities had less administrative meetings for the coordination of the COVID-19 mental health response plan during the second wave.

#### Organizational facilitating factors

##### Reduced delays

With FPs conducting less in-person clinical activity during the pandemic’s first wave, reductions in their levels of mental health referrals to the GASMA for evaluation were highlighted. A decrease in referral requests allowed for a reduction in delays for patient evaluation at some GASMA even with reports of decreased staff availability. For example, a GASMA in one peripheral region aims to treat requests for mental health services within 30 days, an objective according to GASMA participants that could be difficult to meet prior to the pandemic. However, this delay was reduced during the second pandemic wave.

##### Support in the provision of mental health care, including through collaborations

Different types of supports to clinicians were offered. First, psychiatrists shared that they received support from their managers, who worked to offer access to needed technology for teleconsultations (online platforms and computers with cameras, not previously available). In addition, participants shared that they were granted the option of seeing certain patients (unknown to the staff, new evaluations) in-person. Second, the development of lunch-time webinars and meetings for physicians were also useful as the focus was on how to help the general population with limited resources given the pandemic’s impact on health systems. Third, a participant mentioned the extension of the availability of psychosocial aid for healthcare employees, from *“six meetings a year to nine”* (GASMA 2). Last, despite a participant labelling the private sector as *“not magic”* (FP 4) in accessing psychologists during the pandemic context, COVID-19 seemed to *“officialise”* the systematic use of the private sector for *“more fortunate”* patients who had insurance (FP 4).

Intra-organizational collaborations with trained mental health professionals were mentioned. Participants shared that the availability of a social worker with mental health training allowed for quick turnover when there was an emergency and proactive collaboration between the social worker and FP on a *“game plan”* for the patient (FP 3). An FP mentioned the availability of a psychologist, who helped draft a list of available mental health resources where FPs could refer patients during the pandemic. Of note, participants shared that the social worker and psychologist at their respective clinic were available for delivery of mental health care even prior to the pandemic, but their help during the pandemic was instrumental. An FP also shared that during the pandemic, a nurse practitioner with training in mental health care was hired at a university family medicine group and *“help [ed] us with the [mental health] service offer”* (FP 1).

Participants highlighted inter-organizational collaborations. A health manager highlighted the value of meeting with the chief of services from all the health sectors, including mental health, to coordinate health services during the pandemic context. These meetings were rare in the pre-pandemic context. In addition, an FP shared the value of a community of practice among Quebec FPs working in substance use, prior and during the pandemic. Specifically, this community of practice was helpful in issuing *“recommendations, among other things, on how to follow up with patients, and this with methadone or substitution drugs during the pandemic, so [ …] to have a little guidance [on] what can [and can’t be] do [ne]”* (FP 2).

#### Provider facilitating factors

##### Practice characteristics adopted by healthcare professionals

Facilitating mental health practice characteristics included: follow-up of patients with mental health issues (generalized anxiety, anxiety disorders, adjustment disorders) without an FP (“unattached patients”); GASMA workers contacting patients on wait lists *“to provide tools, to direct to organizations that could help”* (health manager 5); *“reaching out”* to patients who are more vulnerable and less likely to seek out mental health services during the pandemic, including by developing and/or mobilizing community-based resources (health manager 4); and leaving FP time slots available for “unattached patients” (i.e., patients without an FP) who consult for mental health services via the emergency department or by self-referrals via a centralized access point (8–1-1 number).

#### Innovation facilitators (technological modalities for service delivery)

##### Technological modalities for mental health service delivery

Participants shared that the pandemic helped Quebec *“propel”* itself into *“computerization”* by developing and/or reinforcing technological solutions for consultations (health manager 3). This development and switch to teleconsultation using online platforms and the increased use of phone consultations helped in *“catching up”* on received mental health requests, including the ones considered *“non-urgent”* (health manager 3). Some GASMA groups resumed by being offered virtually.

Providers appreciated using virtual platforms, including for their efficiency: *“patients are seen quicker, they are not late or things like that”* (psychiatrist 1). Participants shared their perceptions on the utility of phone consultations during the pandemic for elderly patients, patients afraid of the virus, and those *“known”* to the staff, with their mental health condition is considered *“stable”* (FP 5).

### Mental health service delivery recommendations during the pandemic context

Supplementary File 1 highlights the structural and organizational recommendations on mental health service delivery during the first two pandemic waves, as shared by participants. Structural recommendations included ensuring adequate resources for mental health service delivery, specifically by hiring more personnel to respond to mental health need, as well as rethinking access to mental health care by increasing resource allocation at the community level. Participants also shared the need to foster a supportive and welcoming work environment, a way to ensure adequate recruitment of mental health staff including during the pandemic context, as well as to refrain from relocating mental health care staff to *“*COVID-19 *red zones.”* Health service infrastructure recommendations included finding ways to support the general population in times of crises, increasing access to mental health care by including psychological services under the Quebec medical plan, and furthering the implementation of digital technology in Quebec to offer virtual mental health care.

Organizational recommendations centred around inter- and intra-organizational collaborations. For example, participants shared the necessity during the pandemic to change notions of ‘territoriality’ in accessing mental health care, and to support mental health care teams either by replacing staff on medical leave and/or by having a designated team to address mental health specifically in emergency contexts. Intra-organizational collaborations included the need to foster group cohesion in the pandemic context, given shifts from in-person to technological ways of working, as well as increasing the number of available nurses in outpatient hospital clinics to support psychiatrists in mental health care delivery.

## Discussion

This paper explored barriers and facilitators of mental health service delivery in primary care and referral mechanisms during the first two pandemic waves in Quebec. Solutions from the perspective of participants to addressing barriers were also explored. Barriers and facilitating factors were organized according to Chaudoir et al. (2013)‘s framework of structural, organizational, provider- and patient-related, as well as innovation (technological modalities for service delivery) categories [35]. Barriers included ministerial directives on mental health staff relocation to non-mental health related COVID-19 tasks and mental health staff shortage (structural); mental health service interruption (organizational); mental health staff on preventive/medical leave (provider); the pandemic’s effect on consultations (i.e., perceptions of increased demand) (patients); and challenges with the use of technological modalities (innovation). Facilitating factors included reinforcements to mental health care teams (structural); perceptions of reductions in wait times for mental health evaluations during the second wave due to diminished FPs referrals in the first wave, as well as supports (i.e., management, private sector, mental health trained staff) for mental health service delivery (organizational); staff’s mental health consultation practices (provider); and advantages in increasing the use of technological modalities in practice (innovation). Of note, patient-level consequences associated with pandemic and health service organization challenges were captured in the patient barrier section. Participants did not share positive patient factors related to mental health service delivery during the first two pandemic waves, hence the exclusion of a patient facilitating factor section in the results. Recommendations on improving mental health service delivery in Quebec from the perspective of participants included increases in the number of and supports to mental health care staff, and the development of certain mental health infrastructure. Examples of supports to mental health care staff included fostering an attractive work environment to help improve mental health service offer; supporting mental health teams by replacing staff on leave; fostering “group cohesion” in the pandemic context; and offering training to build provider mental health capacity. Including psychological services under the Quebec medical plan and furthering the implementation of digital technology in Quebec were shared examples on how to build mental health infrastructure. Many of the barriers and facilitating factors to mental health service delivery identified by our findings seemed to be consequences of ministerial recommendations for practices to be adopted during the first two phases of pandemic. Therefore, our paper also helped in better understanding the experience participants had with certain of these ministerial recommendations, and their repercussions on mental health service delivery.

Some barriers to mental health service delivery explored in this paper have emerged during the pandemic, while others existed prior and were aggravated. Specific pandemic barriers to mental health service delivery reported by our study included mental health service interruption (e.g., clinic closures or clinic transformation to COVID-19 related establishments, interruption in groups offered in the community, and hospital outpatient clinics). This barrier was also found worldwide during the pandemic context. In a survey conducted by the World Health Organization (WHO), the vast majority (93%) of 130 countries reported mental health service disruptions [36], with outpatient services in mental health and general hospitals, as well as community-based services most significantly interrupted [36]. These service interruptions could make it difficult to uphold some of the Quebec ministerial recommendations suggested to address the anticipated rise in mental health requests during the second pandemic wave, such as increased collaboration between community-based, primary, and specialized care, as well as greater involvement of community-based settings in patient care when appropriate [24]. According to the WHO survey, service disruptions were caused by factors related to both demand (i.e., patients not consulting services) and supply (i.e., redeployment of clinical staff to help with COVID-19 relief, repurposing mental health facilities for COVID-19 initiatives) [36, 37], barriers to mental health service delivery also found in our study. Other barriers identified by our study seem to have been aggravated by the pandemic, such as availability of mental health care staff given their relocation to COVID-19 related tasks, mental health staff being on leave, and clinic closures/transformation to COVID-19 related establishments. In Canada, like in other countries worldwide, shortages of mental health staff have been highlighted as problematic even prior to the pandemic [4, 38]. In November 2020, the Quebec Ministry of Health and Social Services recommended against the relocation of mental health staff and for reinforcement to referral mechanisms like the GASMA should there be a certain level of absenteeism given the essentiality of mental health care during the pandemic in the context of anticipated growing demands for services [22, 23]. Participants’ recommendations also aligned with this ministerial recommendation, by taking the opportunity to think about building back better [39] through investing in publicly funded mental health care with additional human resources like psychiatrists and psychologists, and by placing an emphasis on mental health in primary and community-based settings.

The pandemic’s impact on the general population and populations with pre-existing social and health inequities was mentioned by others [40–45] and was confirmed by our study. In addition, study participants shared that ‘new’ profiles also consulted psychosocial services during the pandemic context, including working parents with children, as well as primary and secondary school students and students enrolled in university. Our findings concord with other studies. First, studies highlight the pandemic’s negative consequences on parents’ mental health [46], as well as female respondents with children under the age of 13 years, the latter reporting higher levels of anxiety and substance use than male respondents [47]. Second, people living alone, and younger adults were more likely to report higher levels of depressive symptoms [2, 3, 44]. Younger adults reported being disproportionately impacted by social isolation and economic challenges [2]. The impact of the pandemic on a wide range of people including those with pre-existing health and social iniquities highlights the need to implement targeted approaches to mental health care delivery [40, 48, 49]. Our study confirms that solutions were developed during the pandemic context to reach people who might not be seeking care (e.g., *“reaching out”* to more vulnerable people by developing/mobilizing community resources and following-up with people on waiting lists), while others used prior to the pandemic were also mobilized to ensure equitable access to mental health care (e.g., FPs’ care and follow-up for “unattached patients”, i.e., patients without an FP). Some of these pandemic facilitators listed by participants were also suggested by the Quebec Ministry of Health and Social Services as recommendations for practice during the second pandemic wave (e.g., a follow-up from the GASMA teams with people whose mental health consultations may have been postponed, who may not have been prioritized, and/or who may be waiting for services) [24].

Some recommendations for mental health service delivery during the pandemic context and shared by study participants should be discussed. First, to ensure the continuity of services during the pandemic context, teleconsultations via online platforms were encouraged and implemented [5, 40, 49–51], also a Quebec ministerial recommendation [22–24] However, as seen by our study, some patients, according to participants interviewed, did not benefit from this type of shift in care, including patients who were of a certain age or did not have an email address and/or material resources to access web platforms. These findings were also confirmed by other studies [5, 52]. Quebec ministerial recommendations during the pandemic context acknowledged this by suggesting that in-person mental health interventions were to be allowed should providers judge it necessary for certain people [24], an organizational facilitating factor identified by study participants. Second, our study highlighted that the private sector’s use for mental health care including psychological services was *“officialized”* during the pandemic context. However, this practice does not benefit patients without private insurance. Interestingly, a participant highlighted that a facilitating factor to mental health service delivery was the increased funding to support mental health teams during the pandemic context. This participant might have alluded to the Quebec government’s investment during the pandemic context: $31.1 million, in part to hire 300 psychologists working in the private sector to offer their services via the public sector, $25 million to buy private sector psychological services for students, as well as $10 million to offer psychological therapy from the private sector to people on wait lists in the public sector [53–56]. However, questions remain as to why this funding for psychological services is merely a product of the pandemic context in Quebec and more largely Canada [56]. Study participants thus shared the need to include psychological services under the Quebec medical plan, also more largely a recommendation prior, during, and post-pandemic in Canada [40, 56].

This study allowed for the exploration of recommendations on improving mental health service delivery in the pandemic context. As shown by our findings (Supplementary File 1), they can be grouped broadly into two areas: 1) strengthening capacity of mental healthcare systems by investing in additional human resources in community and primary care settings, including at referral access points, for patient follow-up and continuity of care, furthering FP mental health training, as well as further implementing digital technologies; and 2) fostering an adaptive health system in emergency settings like pandemics, and this for example by safeguarding mental health services, ensuring access to care (like public psychological services) including through digital modalities, as well as supporting the general population in managing distress and building resilience in times of crises through initiatives like webinars and video capsules on stress management. These recommendations have policy implications, as they can equip policy makers in Quebec, more widely in Canada, and other settings that rely on FPs as gatekeepers to mental health services to respond more effectively to the current and next public health emergency. In fact, recommendations as shared by study participants are also discussed internationally, for example in Europe [57], the Americas [58], and Organisation for Economic Co-operation and Development (OECD) countries [59]. These recommendations may also be used to build back better post-pandemic. With the increasing spotlight on the importance of mental health including in emergency preparedness plans, sharing and implementing these recommendations may help address pre-existing gaps in mental healthcare systems and delivery. Addressing gaps can include the need to further integrate mental health care within primary and community-based settings and improve levels of mental health funding [4, 38].

### Limitations

First, our study sample included health managers and clinicians from three Quebec regions (one healthcare university and two peripheral). While our findings highlight barriers and facilitating factors to mental health service delivery during the first two pandemic waves, as well as recommendations to address these barriers that can be useful across Quebec, these may not be exhaustive. However, the study included regions most impacted by the pandemic (e.g., number of COVID-19 cases and COVID-19 related deaths), and therefore able to provide rich information on the pandemic’s impact on mental health service delivery, and this from a range of actors including health managers. Second, our findings were captured during the first two waves of the pandemic. While they are valuable, other factors may have emerged during subsequent waves. In addition, distinctions between barriers and facilitating factors in mental health service delivery prior to in comparison to during the pandemic, as well as between phases were beyond the scope of this paper. This information would merit further in-depth exploration. Last, in this study, patients were not interviewed on their perceptions of barriers and facilitating factors to mental health service delivery in the pandemic context, as well as their recommendations on addressing these barriers. This information would be useful to better understand what hindered and/or helped people in navigating the healthcare system and receiving mental health care during the pandemic. Patient collaborators included in our project team helped us validate the study findings.

## Conclusion

To our knowledge, this is the first study to explore barriers and facilitating factors to mental health service delivery during the pandemic in Quebec, Canada. Some barriers identified were caused by the pandemic (i.e., relocation of mental health staff to non-mental health related COVID-19 tasks, mental health service interruption, mental health staff on preventive leave), while others seemed to have been entrenched in the mental health system prior to the pandemic, but accentuated (i.e., availability of mental health care staff given their relocation to COVID-19 related tasks, mental health staff being on leave, and clinic closures/transformation to COVID-19 related establishments). Recommendations shared by participants to address barriers to mental health service delivery during the pandemic context highlight the importance of rethinking access to mental health services by: 1) strengthening the capacity of mental healthcare systems by investing in additional human resources in community and primary care settings, including at referral access points, for patient follow-up and continuity of care; and 2) fostering an adaptive healthcare system in emergency settings, for example by safeguarding mental health services and ensuring their availability, including digitally, and offering initiatives like webinars and video capsules on stress management. These recommendations are not only useful during the pandemic for Quebec, Canada, and other contexts that rely on an FP gatekeeper system, but post-pandemic as well to help in further increasing access to mental health care.

## Supplementary Information

**Additional file 1.**

## Data Availability

The datasets generated and analysed during the current study are not publicly available due to confidentiality reasons (i.e., identification of the study participants and study regions) but are available from the corresponding author on reasonable request.

## Abbreviations

FPs: Family physicians
APSS: Programme Accès prioritaire aux soins spécialisés
CRDS: Centres de répartition des demandes de services
GASMA: Guichets d’accès en santé mentale adulte
CIUSSS: Centres intégrés universitaires de santé et de services sociaux
CISSS: Centres intégrés de santé et de services sociaux
PQPTM: Programme québécois pour les troubles mentaux
OECD: Organisation for Economic Co-operation and Development
