# Supplementary material for: Perceptions on barriers, facilitators, and recommendations related to mental health service delivery during the COVID-19 pandemic in Quebec, Canada: a qualitative descriptive study
Source: BMC Prim Care. 2022 Feb 21;23:32. doi: 10.1186/s12875-022-01634-w (PMC8860461; doi:10.1186/s12875-022-01634-w)
Supplement: Supplementary file 1 — Additional file 1. [file 12875_2022_1634_MOESM1_ESM.docx]

Supplementary File 1: Mental health service delivery recommendations from the perspective of participants

| **Factor** | **Recommendations** | **Illustrative** |
| --- | --- | --- |
| **Structural** | HUMAN RESOURCES  1. Ensuring adequate mental health resources  2. Fostering *“an attractive”* work environment to help improve mental health service offer  3. Refraining from relocating mental health care staff  HEALTH SERVICE INFRASTRUCTURE  4. Supporting the general population to help manage distress in times of crises  5. Including psychological services under the Quebec medical plan  6. Furthering the implementation of digital technology in Quebec | **Hiring more personnel to respond to mental health demand in primary and community-based settings, as well as psychiatrists:**  *“More staff should be hired to respond to first-line follow-ups and more psychiatrists […] at our level [GASMA] […].”* (*Guichets d’accès en santé mentale adulte* (GASMA) 4)  **Rethinking access to mental health care with more community resources:**  *“I think it's just a social issue, there are a lot of articles that came out during the pandemic just like what it is a social issue and that we had to rethink our system of access to mental health, increase resources at the community level […]”* (family physician (FP) 6)  **Increasing psychologist effective to respond to specific patient needs:**  *“[…] there are cases where I think it really takes someone who is trained in psychology to give tools to patients who have […] anxiety disorders […] and right now we don't have access to a psychologist. We don't have any. We have almost zero.”* (FP 5)  *“[…] that we be more attractive to new employees, that people want to come work with us, that we keep those who are there too. So it's to have a pleasant context, but knowing that we cannot have time off, are overwhelmed, can catch COVID, be relocated to places that we do not want to go to, it is not attractive for people, then people leave […] or they don't apply.”* (GASMA 1)  *“It does not make sense for mental health, to relocate, and I understand that the need is there, but the need is also very much in mental health and we must support people at this time [but] it's easy to judge, I don't know, if I was the Ministry, what would I have done for real […] but I find that the mental health field should not have been touched.”* (GASMA 2)  *“Can we do group sessions, webinars on stress management in a pandemic, things like that, for ‘Mr. and Mrs. Everybody,’ […] [so] we can reach more people. Video capsules, video clips, things like that, to have advertising on TV, I think there are other ways to reach people rather than sitting face to face in an office.”* (FP 4)  *“[…] a psychologist should be part of universal health care; we pay for pills […] I don't understand why we don't pay for psychologists.”* (FP 5).  *“The next step is to say faxes, for real in 2020, faxes or pagers, it's just the health care network using them, that's ridiculous […] there is no more reason to have that, we have secure email addresses. […] A big, big step was all the teleconsultation, it has been deployed a lot [but] there is one more step to be taken in my opinion, that we eliminate it [the fax] completely and really move on to all the computerization”* (health manager 3) |
| **Organizational** | INTER-ORGANIZATIONAL COLLABORATIONS  1. Changing the notion of *“territoriality”* in mental health care offer  2. Supporting mental health teams by replacing staff on leave  3. Having a crisis/emergency team to address mental health issues in a timely fashion.  INTRA-ORGANIZATIONAL COLLABORATIONS  4. Fostering *“group cohesion”* in the pandemic context  5. Better supporting psychiatrists in outpatient hospital clinics with nurses for collaborations  HEALTH SERVICE ORGANIZATION  6. Resuming services that were interrupted, like groups  7. Offering training to build FPs’ mental health capacity | *“When we have patients who are a little more complex and who we would like to send for example to more specialized hospitals, because […] we are a generalist department, we are not an institute, [the specialized hospital] says "Oh well no, the patient is not from our territory, we cannot give him services, because we are not funded for him". […]. So, all these notions of territory, geographical affiliation, and accessibility, if the COVID could allow us to think about that and to change this mode of operation, I would be very happy.”* (psychiatrist 1)  *“[…] let's say from May, June, July [2020], […] about half the staff were missing, but during that time, we said to each other ‘okay, the people are temporarily not here, but to have a kind of […] standby team like there is in the hospital to come and lend a hand for a while to avoid there are too many delays.”* (GASMA 1)  *“[…] in times of pandemic or crisis situation, it would almost take another team […] made up of doctors, [psychosocial] workers […] a team dedicated to crisis situations, which can be deploy or even move, depending on the location and take charge of people without necessarily having a referral. Possibly do a first triage […] for people who are really in distress and who refer them to a psychiatrist or not, but […] without delay […] it has to be done quickly […] you don't have to go through […] the family doctor who will make a referral, who will speak to the healthcare provider and the psychiatrist afterwards.” (FP 7)*  *“However, in the context of a pandemic, as team, as people who have had to adapt to remote working, to working a little more isolated from each other, well there is really something that needs to be done […] we have tried to maintain team cohesion, a team bond, team support because if physically we are not all together, to help each other and to support each other, if we are remote working, well it's more difficult.”* (health manager 5)  *“[…] that we be better supported in the outpatient clinic […] if I could work closely together with a nurse, I could be more productive, see more new cases, maybe the nurse could do follow-ups and we work together for follow-up appointments […] so it might free my time up for new cases. There is currently one nurse for three psychiatrists.”* (psychiatrist 3)  *“We have abandoned activities, I would like them to start again […]. For example, we had group therapy for anxiety disorders, for bipolar disorders, for personality disorders; that no longer exists with the pandemic, so it is certain that our service offer has diminished.”* (psychiatrist 2)  *“[…] another thing that could possibly improve […] we should see to the training of general practitioners who are interested in mental health [as] they could be useful in primary care or in the settings in which they work.”* (FP 7) |
